# Bioactive Glass Inhibits Tumor Development from Giant Cell Tumor of Bone-Derived Neoplastic Stromal Cells in a Chicken Chorioallantoic Membrane Assay

**DOI:** 10.3390/cancers15061868

**Published:** 2023-03-20

**Authors:** Joerg Fellenberg, Sarina Losch, Max R. Marinescu, Birgit Frey, Burkhard Lehner, Marcela Arango-Ospina, Zoya Hadzhieva, Aldo R. Boccaccini, Fabian Westhauser

**Affiliations:** 1 Experimental Orthopaedics, Department of Orthopaedics, Heidelberg University Hospital, 69118 Heidelberg, Germany; 2 Department of Materials Science and Engineering, Institute of Biomaterials, University of Erlangen-Nuremberg, 91058 Erlangen, Germany

**Keywords:** giant cell tumor of bone, bioactive glass, chorioallantoic membrane assay

## Abstract

**Simple Summary:**

High recurrence rates represent a major problem during the management of giant cell tumors of bone (GCTB). To evaluate the suitability of bioactive glasses (BGs) for the development of new therapeutic strategies, we analyzed the effects of BGs on cell viability and tumor formation. We observed a strong cytotoxic effect of BGs toward primary GCTB cell lines in vitro and a significant reduction of tumor formation and growth using a chicken chorioallantoic membrane (CAM) assay. These data suggest that BGs represent promising candidates for the treatment of GCTBs.

**Abstract:**

Tumor recurrence is a major problem during the treatment of giant cell tumors of bone (GCTB). We recently identified tumor cell-specific cytotoxic effects of bioactive glasses (BGs) toward neoplastic stromal cells derived from GCTB tissue (GCTSCs) in vitro. Since these data indicated a promising role of BGs in the adjuvant treatment of GCTBs, we aimed to investigate the transferability of the in vitro data into the more complex in vivo situation in the current study. We first analyzed the cytotoxicity of three different BGs in vitro by WST-1 assay after co-cultivation with primary GCTSC cell lines. The effects of BGs on tumor engraftment and growth were analyzed by chicken chorioallantoic membrane (CAM) assays and subsequent quantification of tumor take rates and tumor volumes. In vitro, all tested BGs displayed a cytotoxic effect on GCTSCs that was dependent on BG composition, concentration, and particle size. Comparable effects could be observed within the in vivo environment resulting in reduced tumor take rates and tumor volumes in BG-treated samples. These data indicate a possible clinical application of BGs in the context of GCTB therapy, mediating a reduction of recurrence rates with the simultaneous promotion of bone regeneration.

## 1. Introduction

Giant cell tumors of bone (GCTBs) are semi-malignant bone tumors that predominantly occur in young adults aged 20–30 years and are most frequently located in the distal femur, proximal tibia, or distal radius [1]. They account for approximately 5% of all primary bone tumors and are associated with expansive osteolytic defects, infiltration of the surrounding tissue and bone destruction [2,3]. On the cellular level, GCTB consists of stromal cells (GCTSC), multinucleated giant cells, and giant cell precursor cells. GCTSCs represent the neoplastic cell population and induce the formation of bone-destructive giant cells by expression and secretion of the receptor activator of NF-κB ligand (RANKL), a key mediator of osteoclast activation. The osteoclast-like giant cells are finally responsible for the formation of the characteristic osteolytic lesions found in GCTB.

There are mainly two different options to treat GCTB, namely en bloc resection of the tumor, including the adjacent joints or intralesional curettage [4,5]. While the latter is considered to be the standard therapy providing better functional outcomes compared to en bloc resection [4], it is, however, associated with very high recurrence rates (20–65%). The use of adjuvants such as hydrogen peroxide, ethanol, or liquid nitrogen can further influence the degree of tumor recurrence [6,7,8]. To improve the therapy outcome, bisphosphonates [9] and denosumab, a monoclonal anti-RANKL antibody [10], have already been used for GCTB treatment. However, the neoplastic stromal cell population that is responsible for tumor recurrence is not targeted by these therapies, and denosumab has further been shown to increase local recurrence rates [11] and may be associated with malignant transformation [12]. Thus, new therapeutic approaches that effectively reduce recurrence rates still have to be developed. Besides the reduction of tumor recurrence, bone regeneration is an important issue in the course of GCTB management. Tumor growth and surgical intervention both lead to the generation of bone defects that have to be treated [6,13]. Poly methyl methacrylate (PMMA) and cancellous bone are frequently used to fill larger bone defects. PMMA is characterized by high mechanical stability [6] and has been shown to decrease recurrence rates, but it is biologically inactive and will thus not provide biological regeneration of the bone [14]. Cancellous bone, on the other hand, has been shown to be associated with high local tumor recurrence rates (65% vs. 35% in the case of PMMA). Attractive alternatives for the treatment of bone defects are bioactive glasses (BGs) [15]. The first representative of this group of materials, named 45S5-BG, was originally developed by Hench et al. in the late 1960s [16]. In physiological solutions, BGs develop a hydroxyapatite layer on their surface that provides a strong bonding and a stable integration into the surrounding bone tissue [17,18]. Depending on their composition, BGs can further stimulate various cellular functions, including osteogenic differentiation of precursor cells mediated by the release of ionic dissolution products [19,20]. In addition to their already existing broad clinical application in areas such as regenerative medicine, dentistry, and the treatment of infections [21], BGs may further enable a wide range of applications for the treatment of tumors. They have been shown to offer promising approaches for new therapeutic strategies, including the synthesis of magnetic BGs for hyperthermia therapy, the use of BGs as a delivery system for anticancer drugs, metal ions, or small molecules, and the induction of apoptosis [22,23,24]. Interestingly, we recently identified cytotoxic effects of BGs that were mostly affecting GCTB tumor cells but not non-malignant cells such as mesenchymal stromal cells (MSCs). In addition to that, the proliferation and viability of MSCs were even enhanced by the presence of BGs. Since MSCs are of key relevance for bone defect regeneration, these data indicate that BGs represent promising biomaterials that might be used to target both tumor recurrence and bone regeneration [25,26]. So far, these results are limited to two-dimensional cell culture experiments that lack the more complex influences within a three-dimensional tumor matrix and microenvironment. Therefore, it remains unclear if the observed effects of BGs on GCTSCs can be reproduced in an in vivo environment. However, these data are relevant to assess whether the proposed synergistic mechanism of a reduction of tumor recurrence and enhancement of bone regeneration holds true and might be transferable to the clinical treatment of GCTBs in the future. To verify this hypothesis and to evaluate the suitability of BGs for the development of new therapeutic strategies that simultaneously target tumor recurrence and bone regeneration, we aimed to analyze the effects of BGs on tumor formation and growth in the in vivo environment of a chicken chorioallantoic membrane (CAM) assay in this study.

## 2. Materials and Methods

### 2.1. Synthesis of Bioactive Glasses

Synthesis of bioactive glasses was performed using the melt-quenching method as described previously [26]. Sintering of 45S5-BG was carried out at 1050 °C for 2 h, while ICIE16-BG and 3Zn-BG were sintered for 1.5 h at 690 °C. BG compositions are presented in Table 1. A jaw crusher (Retsch, Haan, Germany) was used for crushing the BGs before they were ground to powder using a planetary mill (Retsch, Haan, Germany) and sieved to the desired particle sizes. The software ImageJ (National Institutes of Health Bethesda, MD, USA) was used to estimate the particle sizes on the basis of SEM (scanning electron microscopy) images. Further characterization of BGs was performed as previously described in detail [25].

### 2.2. Isolation and Culture of GCTSC

GCTSC were isolated from tumor tissue that was cut into small pieces using scalpels, washed with PBS, and digested with 1.5 mg/mL collagenase B (Thermo Fisher Scientific, Dreieich, Germany) for 3 h at 37 °C. After digestion, the cell suspension was pushed 10 times through an 18 gauge needle to separate cells, washed twice in PBS, and seeded in a culture medium consisting of DMEM high glucose supplemented with 10% FCS (Biochrom, Berlin, Germany) and 100 U/mL penicillin/streptomycin (Sigma-Aldrich, Taufkirchen, Germany). Cells were split after 24 h and cultured for three further passages to remove unwanted giant cells and histiocytes.

### 2.3. Cell Viability Assay

For quantification of BG-mediated cytotoxicity BGs and cells (10.000 per well) were mixed and plated into 96-well culture plates. After the desired incubation times, the culture medium was replaced by WST-1 reagent (Santa Cruz, Heidelberg, Germany) diluted 1:10 in the culture medium, and the samples were further incubated for 120 min at 37 °C. The color change resulting from the conversion of the substrate was quantified at 450 nm using a microplate reader (Auto-Phomo, Anthos Microsystems, Friesoyte, Germany). A reference wavelength of 600 nm was used, and the value of blank wells was subtracted from the experimental samples. All measurements were performed in triplicates.

### 2.4. Chicken Chorioallantoic Membrane (CAM) Assay

Tumor growth was analyzed on the chorioallantoic membrane of fertilized chicken eggs that were obtained from a local hatchery (Geflügelzucht Hockenberger, Eppingen, Germany). Upon arrival, eggs were cleaned with dry paper towels and incubated at 37.8 °C, 70% humidity, and permanent agitation. After three days, 3 mL of albumen was removed with a syringe before a window was cut into the eggshell and resealed with Durapore tape (3M, Neuss, Germany). This opening was then used on day 9 to transplant the cells onto the chorioallantoic membrane. For transplantation, cells were resuspended in DMEM culture medium (1 × 10^6^ cells/20 µL) containing the BGs of interest in the desired concentration and mixed with an equal volume of Cultrex BME Type 3 (AMS Biotechnology, Frankfurt, Germany) that served as matrix and nutrient supply. A silicone ring was applied to the CAM through the window, and the CAM within this ring was gently lacerated to promote subsequent vascularization. After transplantation of 40 µL cell-matrix suspension into the center of the silicone ring, eggs were closed and incubated for further seven days. On day 16, embryos were euthanized using the pentobarbital Narcoren before the tumors were removed. Tumor take rates were calculated as the number of eggs with tumors × 100/number of eggs with vital embryos. The tumor volumes were calculated using the following formula: volume = 4/3 × pi × r3 (r = ½ × √ of diameter 1 × diameter 2) [27]. At least 20 eggs were used for each experimental group.

### 2.5. Lens Culinaris Agglutinin Staining

Formalin-fixed and paraffin-embedded tumor tissue was used for the detection of chicken cells and blood vessels. Sections were deparaffinized using Roti-Histol (Carl-Roth, Karlsruhe, Germany) and rehydrated with isopropanol (100%, 96%, 70%, and 50%, 5 min each). After 15 min of blocking with 5% bovine serum albumin (BSA), samples were incubated with 5 µg/mL biotinylated lens culinaris agglutinin (Linaris, Dossenheim, Germany) diluted in PBS for 30 min at room temperature in a wet chamber. Sections were washed twice with PBS supplemented with 0.5% Tween 20 and once in PBS without supplements before they were incubated for 30 min in AB reagent (Avidin D/biotinylated alkaline phosphatase) (Linaris, Dossenheim, Germany). Sections were washed as described above and incubated with ImmPACT Vector Red (Linaris, Dossenheim, Germany) alkaline phosphatase substrate. After 20 min, staining was stopped by immersion in water before sections were counterstained with methyl green (Linaris, Dossenheim, Germany), mounted with NeoMount (Merck-Millipore, Darmstadt, Germany), and photographed using a Keyence BZ-X800 microscope (Keyence, Neu-Isenburg, Germany).

### 2.6. ALU In Situ Hybridization

Formalin-fixed and paraffin-embedded tumor tissue sections were deparaffinized using Roti-Histol (Carl Roth, Karlsruhe, Germany) and rehydrated in isopropanol. Antigen retrieval was performed by digestion of the tissue with (5 µg/mL) proteinase K (Roche Diagnostics, Mannheim, Germany) for 15 min at 37 °C. Before hybridization, sections were pretreated with 0.1 M triethanolamine (pH 8.0) supplemented with 0.25% acetic acid for 10 min on a stirrer. Prehybridization was performed at 42 °C using a hybridization buffer consisting of 4 × SSC (saline sodium citrate), 50% deionized formamide, 1 × Denhardt’s solution, 5% dextrane sulfate, and 100 µg/mL salmon sperm DNA. After 60 min, the prehybridization solution was replaced by fresh hybridization buffer supplemented with 0.2 ng/µL heat-denatured digoxigenin-labeled ALU probe, synthesized by PCR as described previously [28]. After hybridization for 16 h at 42 °C slides were washed twice in 2 × SSC buffer for 5 min at room temperature and twice in 0.1 × SSC for 10 min at 42 °C. Finally, a bound ALU probe was visualized using anti-Digoxigenin alkaline phosphatase-conjugated Fab fragments (Roche Diagnostics) and NBT/BCIP (Linaris, Dossenheim, Germany) as substrate. Sections were counterstained with methyl green (Linaris, Dossenheim, Germany), mounted with NeoMount (Merck-Millipore, Darmstadt, Germany), and photographed using a Keyence BZ-X800 microscope (Keyence, Neu-Isenburg, Germany).

### 2.7. H3.3-G34W Immunohistochemistry

Formalin-fixed and paraffin-embedded tumor tissue was deparaffinized in Roti-Histol (Carl Roth) and rehydrated in isopropanol before sections were incubated in a pressure cooker at 121 °C for 5 min in a coplin jar containing Dako target retrieval buffer pH 6 (Dako, Hamburg, Germany). Sections were cooled down to room temperature, blocked for 15 min in PBS supplemented with 5% BSA and incubated overnight at 4 °C with a rabbit anti-H3.3 G34W antibody (RevMab Biosciences, San Francisco, CA, USA) diluted 1:200 in PBS supplemented with 1% BSA. Signals were detected using a BrightVision plus kit (VWR International, Bruchsal, Germany) according to the manufacturer’s instructions. For visualization, the red substrate ImmPACT Vector Red (Linaris, Dossenheim, Germany) was used. After 30 min of staining, the samples were counterstained with methyl green, mounted with NeoMount (Merck-Millipore, Darmstadt, Germany), and photographed using a Keyence BZ-X800 microscope (Keyence, Neu-Isenburg, Germany).

### 2.8. Statistics

SPSS software (IBM, Armonk, NY, USA) was used for the statistical evaluation of the data. The different experimental groups were compared using a Mann–Whitney U test. Calculated *p*-values are two-sided, with *p*-values < 0.01 considered as highly significant and *p* < 0.05 as significant.

## 3. Results

To verify our recent findings showing selective cytotoxicity of BGs toward GCTSCs [25,26], we treated GCTSCs (*n* = 5) with different BGs and analyzed cell viability by WST-1 assay in vitro. Besides 45S5-BG, which is already in clinical use as a bone substitute material, we analyzed the closely related ICIE16-BG, which is characterized by reduced sodium but increased calcium content, and 3Zn-BG, a modification of ICIE16-BG, with increased zinc content (Table 1). If not otherwise stated, a sintering process was included during the synthesis of all BGs used in this study.

### 3.1. Cytotoxic Effects of BGs In Vitro

**Figure 1:**
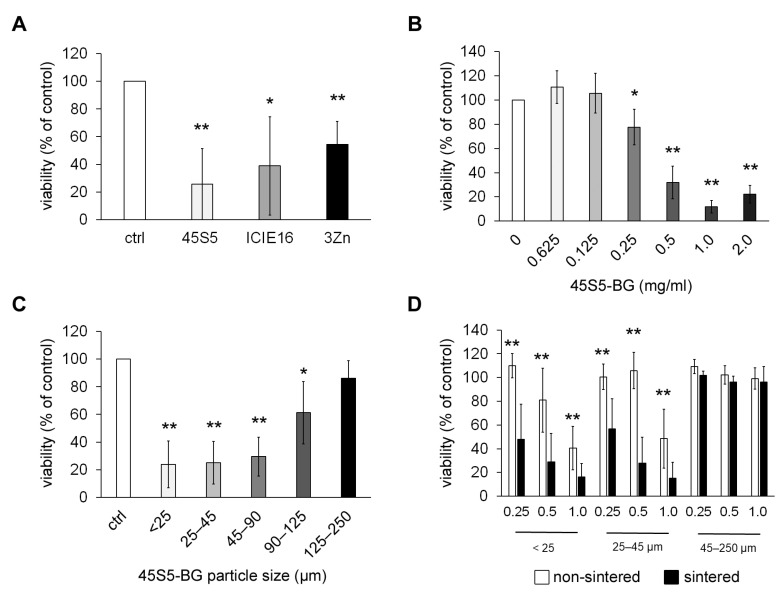
Cytotoxic effects of BGs in vitro. (**A**) GCTSCs (*n* = 5) were co-cultured with the indicated BGs at a concentration of 0.5 mg/mL for 72 h. Viability was analyzed by WST-1 assay and is expressed as percent viability compared to untreated control cells. (**B**) GCTSCs (*n* = 5) were co-cultured with 45S5-BG for 72 h at the indicated concentrations before cell viability was analyzed by WST-1 assay. (**C**) GCTSCs (*n* = 5) were co-cultured with sintered 45S5-BG with the indicated particle sizes at a concentration of 0.5 mg/mL for 72 h. Cell viability was quantified by WST-1 assay. (**D**) GCTSCs (*n* = 5) were co-cultured with sintered and non-sintered 45S5-BG, respectively, at the indicated concentrations and particle sizes. Cell viability was analyzed after 72 h. (* *p* < 0.05, ** *p* < 0.01).

All tested BGs induced a significant reduction of cell viability after co-cultivation with tumor cells, with 45S5-BG showing the strongest effects (Figure 1A). Therefore, 45S5-BG was used for further experiments demonstrating a concentration-dependent induction of cell death that was already significant at a concentration of 0.25 mg/mL and was most pronounced at 1.0 mg/mL (Figure 1B). In addition to this confirmation of the previously identified cytotoxic effects of BGs on GCTSCs, we further analyzed the importance of BG particle size and surface properties for BG-mediated cell death. The 45S5-BG batch used in this and our previous experiments was a mixture of different particle sizes ranging from 45 to 85 µm. Since we recently observed that direct cell–BG contact is necessary for the induction of cell death [26], we concluded that not only the BG composition but also physical parameters may be important to mediate cytotoxicity. We, therefore, analyzed the effects of different 45S5-BG particle sizes and observed that the extent of cytotoxicity is significantly influenced by particle size, with smaller particles being more effective (Figure 1C).

The sintering process performed during BG synthesis influences the BG surface due to partial crystallization that might occur in the material when it undergoes a high-temperature thermal treatment. In the case of 45S5 BG, this process starts after 700 °C with the formation of combeite phases. The effects of two different crystallization processes on BGs have been reported, namely, surface and bulk crystallization, with surface crystallization being the dominant mechanism on 45S5 BG particles of less than 45 µm, whereas for coarse particles (300–500 µm) and millimeter-sized plates, bulk crystallization was found to be the driving mechanism. Furthermore, complete densification of BG particles can be affected or inhibited by partial crystallization of the material, which might lead to pores and inhomogeneous dissolution of the glass. Due to these extensive changes in the BG surface, we further compared the cytotoxic effect of sintered and non-sintered BGs in three different size fractions. Again, smaller particles were much more effective concerning the reduction of cell viability. Interestingly, sintering significantly enhanced this effect (Figure 1D).

### 3.2. Cytotoxic Effects of BGs in CAM Assays

The main aim of this study was to investigate whether the described in vitro effects can also be detected under in vivo conditions. For this, we used a chicken chorioallantoic membrane (CAM) assay that we previously optimized for use with osteosarcoma cells [28]. Per egg, 1 × 10^6^ primary GCTSCs in 20 µL culture medium were mixed with an equal volume of Cultrex BME Type 3, a soluble form of basement membranes that served as a matrix. This matrix is necessary to immobilize the cells on the CAM after transplantation and to supply the cells with nutrients within the first 72 h. The cell-matrix suspension was transplanted onto the CAM of fertilized chicken eggs. After seven days, tumors that had formed were resected, photographed, and analyzed.

#### 3.2.1. Detection of Human H3.3-G34W-Positive Cells in Resected Xenografts

**Figure 2:**
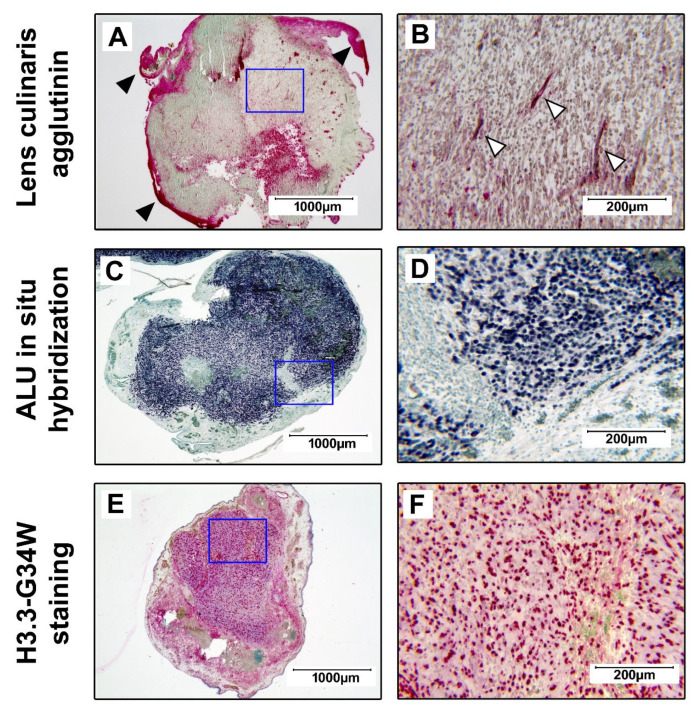
Detection of human H3.3-G34W-positive stromal cells in tumor xenografts. The resected tumor tissue was fixed in 4% paraformaldehyde, embedded in paraffin, sectioned, and stained. (**A**,**B**) Lens culinaris agglutinin staining showing the red stained CAM (▶) and blood vessels (▷) within the unstained tumor tissue. (**C**,**D**) Hybridization of the xenograft with a probe specific for the human ALU DNA sequence (dark blue). (**E**,**F**) H3.3-G34W expression in GCTB stromal cells (red). The blue frames define the inserts shown at a higher magnification on the right. All samples were counterstained with methyl green.

Before we investigated the effects of BGs on tumor development, we aimed to characterize the tumor tissue that formed after the transplantation of GCTSCs on the CAM. We initially performed a lens culinaris agglutinin staining with paraffine-embedded xenografts. With this method, chicken cells within the CAM and the blood vessel-forming endothelial cells can be visualized while human cells remain unstained. We observed that all resected tumors consisted of unstained cells with scattered blood vessels, surrounded by strongly stained chicken CAM cells (Figure 2A,B). In the next step, we performed an ALU in situ hybridization specific for the human ALU restriction site that confirmed the human origin of the cells forming the tumor mass (Figure 2C,D). We could further prove the expression of mutated H3.3-G34W within the tumor xenografts, a characteristic feature of neoplastic GCTB stromal cells (Figure 2E,F).

#### 3.2.2. Influence of BG Concentration on Tumor Formation

**Figure 3:**
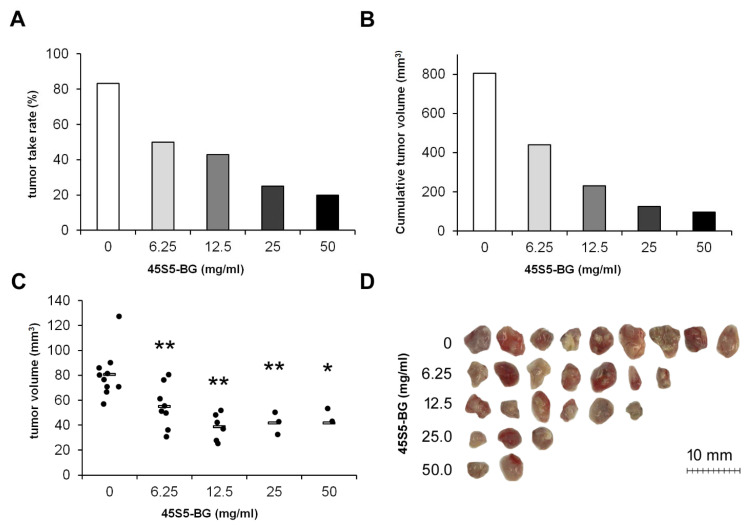
Concentration-dependent inhibition of tumor growth by 45S5-BG. GCTSC (1 × 10^6^ per egg) were combined with 45S5-BG at the indicated concentrations and transplanted onto the CAM of fertilized chicken eggs (*n* ≥ 20 per experimental group). After seven days, tumors that had formed were resected, and (**A**) the tumor take rate, (**B**) the cumulative tumor volumes, and (**C**) the individual tumor volumes were calculated. (**D**) Photographs of the resected tumors. (* *p* < 0.05, ** *p* < 0.01 compared to the control group without BG).

We next investigated whether BGs are able to inhibit tumor growth within the in vivo environment provided by the CAM and which BG concentration is necessary to achieve a significant effect. Based on our in vitro data, we only used sintered BGs for these experiments. If not otherwise stated, the particle size of the 45S5-BG, ICIE16-BG, and 3Zn-BG granules was 65 ± 20, 62 ± 27, and 43 ± 19 µm, respectively, as estimated earlier from scanning electron images [25]. To investigate the influence of the BG concentration on tumor formation GCTSCs were mixed with 45S5-BG at a concentration of 6.25, 12.5, 25, and 50 mg/mL together with Cultrex BME Type 3 matrix. The cell-matrix-BG suspension was transplanted onto the CAM of fertilized chicken eggs. After seven days, tumors that had formed were resected, and the tumor take rates and the tumor volumes were calculated. Tumor take rates were calculated as: number of eggs with tumors × 100/number of eggs with vital embryos. While untreated cells formed solid tumors in 83% of all cases, 45S5-BG reduced the tumor take rate to 20% when used at 50 mg/mL (Figure 3A). As a consequence, the cumulative tumor volume markedly decreased from 805 mm^3^ to 96 mm^3^ (Figure 3B). The individual tumor volumes significantly decreased from 80.6 mm^3^ in the control group to 41.7 mm^3^ after treatment with 50 mg/mL 45S5-BG (Figure 3C,D).

#### 3.2.3. Influence of BG Particle Size on Tumor Formation

**Figure 4:**
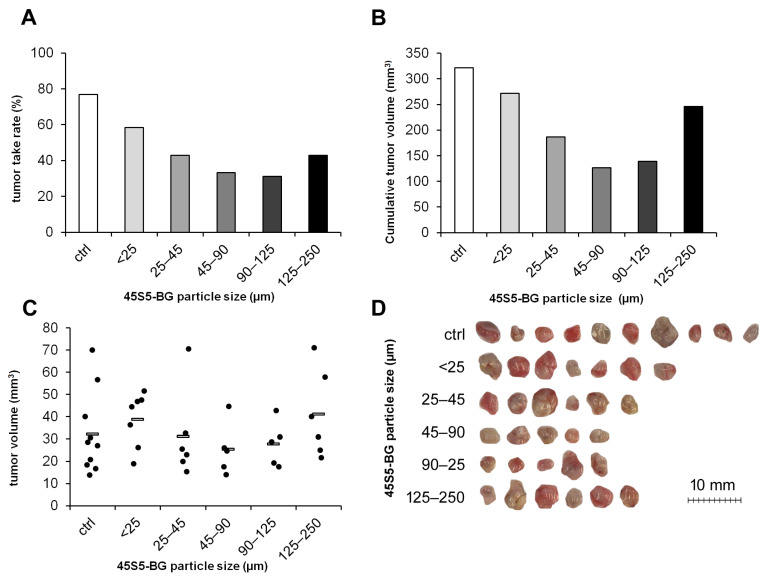
Effect of 45S5-BG particle size on tumor formation and growth. GCTSC (1 × 10^6^ per egg) were combined with 45S5-BG with the indicated particle sizes at a concentration of 25 mg/mL and transplanted onto the CAM of fertilized chicken eggs (*n* ≥ 20 per experimental group). After seven days, tumors that had formed were resected, and (**A**) the tumor take rate, (**B**) the cumulative tumor volumes, and (**C**) the individual tumor volumes were calculated. (**D**) Photographs of the resected tumors.

We next investigated whether the particle size also affects the degree of cytotoxicity in the in vivo environment. GCTSCs were mixed with 45S5-BG particles at a concentration of 25 mg/mL ranging in size from <25 µm up to 250 µm. All particle sizes tested inhibited tumor formation, with particles ranging from 45 to 90 µm being the most effective. These particles reduced the tumor take rate from 77% in the untreated control group to 33% and the cumulative tumor volume from 322 mm^3^ to 126 mm^3^ (Figure 4A,B). Although not significant, the individual tumor volumes were lowest in the group with BG particle sizes ranging from 45 to 90 µm (Figure 4C,D).

#### 3.2.4. Influence of BG Composition on Cytotoxicity

**Figure 5:**
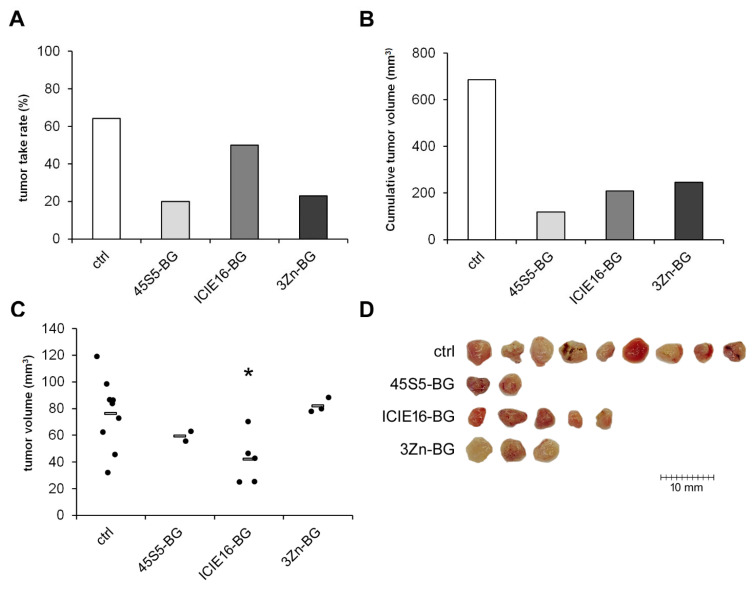
Effect of different BG variants on tumor formation and growth. GCTSC (1 × 10^6^ per egg) were combined with 45S5-BG, ICIE16-BG, or 3Zn-BG at a concentration of 25 mg/mL and transplanted onto the CAM of fertilized chicken eggs (*n* ≥ 20 per experimental group). After seven days, tumors that had formed were resected, and (**A**) the tumor take rate, (**B**) the cumulative tumor volumes, and (**C**) the individual tumor volumes (**C**) were calculated. (**D**) Photographs of the resected tumors (* *p* < 0.05 compared to the control group without BG).

Inhibition of tumor growth could be achieved with all BG variants tested (45S5-BG, ICIE16-BG, and 3Zn-BG); however, effectiveness varied with the chemical composition. Tumor take rates were reduced from 64% in the control group to 20%, 50%, and 23%, respectively. The cumulative tumor volumes decreased accordingly from 686 mm^3^ in the control group to 118 mm^3^, 209 mm^3^, and 245 mm^3^ (Figure 5A,B). Although ICIE16-BG showed the least effect on the tumor take rate compared to the other BGs, the individual tumor volumes were significantly lower compared to the untreated control group. (Figure 5C,D).

## 4. Discussion

Tumor recurrence is a major problem in the context of GCTB treatment. It is greatly influenced by several factors, including the surgical technique ranging from intralesional curettage to wide resection, the use of bone fillers such as PMMA or cancellous bone, and the application of adjuvants including liquid nitrogen, hydrogen peroxide, and alcohol [29,30,31]. To date, surgical removal is regarded as the gold standard for the therapy of GCTB; however, the effectiveness of adjuvants that have been proven to suppress the high recurrence rates associated with this procedure is still not sufficient. Based on our previous observations showing specific cytotoxic effects of BGs toward GCTSCs in vitro and their known osteogenic properties, we assumed that BGs might represent a promising option for the effective reduction of tumor recurrence together with a simultaneous promotion of bone regeneration [26]. In a previous study, we observed a MAPK (mitogen-activated protein kinase)-dependent expression of certain transcription factors and subsequent induction of autophagy in BG-treated GCTSCs that was not seen in mesenchymal stromal cells (MSCs) that were analyzed as controls [25]. However, the precise mechanism of tumor cell specificity is not yet clear. Interestingly, the cytotoxic effect of BGs seemed to be dependent on direct contact of cells with the BGs and could not be mediated by transwell experiments or conditioned media. These data indicated that the release of ions from the BGs into the cell culture medium and the associated change of the pH value is not sufficient for the induction of cell death. The need for direct contact between cells and BGs rather suggests that physical parameters such as particle size or surface properties might be important. In fact, we observed that in vitro, the BG-induced cell death depends on the composition of the BG, the BG concentration, the BG particle size, and the implementation of a sintering process during BG synthesis. The latter suggests an important influence of the BG surface on the induction of cell death and a potential role of cell adhesion molecules such as cadherins and integrins during this process. However, since in vitro studies represent an artificial system that cannot be transferred directly into the in vivo situation, we aimed to verify our in vitro data within an in vivo setting also to enhance the translational character of our approach. We have chosen the CAM assay for these investigations since it is a well-established model system that represents a link between in vitro experiments and more complex in vivo models and further supports the replacement, reduction, and refinement of animal experiments according to the 3Rs principle. CAM assays have already been used to study the development and progression of various tumors, including prostate cancer [32], glioblastoma [27,33], neuroblastoma [34], squamous cell carcinoma [35], and others. The CAM represents an ideal substrate for tumor growth since it is highly vascularized and mimics, together with extracellular matrix proteins, the physiological tumor environment. Grafting of tumors is further facilitated by a natural immunodeficiency of the CAM lacking cell-mediated immunity until day 14 [36]. Further advantages of the CAM assay compared to classical animal models are high reproducibility, cost-effectiveness, and short incubation times [37].

Using this approach, we could verify the cytotoxic effects of BGs on tumor cells also in this in vivo situation, resulting in reduced tumor take rates and tumor volumes. The observed effects were dependent on the BG composition, BG concentration, and particle size. Tumor growth could be inhibited with all BGs tested, with 45S5-BG being the most effective. Compared to 45S5-BG, ICIE16-BG is characterized by a replacement of the majority of sodium ions by calcium and potassium as well as a reduced phosphorus concentration. 3Zn-BG, on the other hand, is an ICIE16-BG variant that additionally contains zinc (Table 1). These changes in the BG composition, however, did not lead to an increase in the inhibitory effect on tumor growth but rather to a reduction, suggesting an important role of the sodium ions that have been replaced in ICIE16-BG and 3Zn-BG. Our observation that the inhibitory effect of BGs on tumor growth increased with the BG concentration might be explained by elevated ion concentrations or more pronounced changes in the pH value. However, since we observed in vitro that a cell-BG contact is necessary for the cytotoxic effect, the increase in total BG surface area might also be significant. This might also explain our observation that when used in equal amounts, smaller BG particles with a larger surface area are more effective than bigger ones, at least in vitro. In the CAM assays, there seems to be an optimum particle size ranging from 45 to 90 µm. Potentially, due to the longer incubation times compared to the in vitro experiments, the inhibitory effect of BG particles on tumor formation is reduced by degradation processes that should be most noticeable for the smaller fragments. Indeed, it has already been shown that the rates of BG dissolution are higher in simulated body fluid compared to cell culture medium and increase with decreasing particle size [38]. These observations indicate that further studies are needed to determine the optimal chemical and physical properties of BGs to maximize their cytotoxic effect on tumor cells. In addition, our observation that direct contact of cells and BGs is necessary for the BG-mediated cytotoxic effect might be a limitation of our proposed therapeutic strategy in vivo since tumor cells hiding in bone cavities might not be affected and may thus trigger tumor recurrence. Thus, it has to be determined whether direct BG-cell contact is a basic requirement for BG-mediated cytotoxicity also in vivo or if BG dissolution products are sufficient to mediate the cytotoxic effects in this setting.

## 5. Conclusions

We detected strong inhibitory effects of various BGs on tumor formation and growth within an in vivo setting. Our data prove that BG-mediated selective cytotoxicity against GCTSCs is not limited to two-dimensional cell culture settings but is also effective in more complex in vivo situations. Furthermore, we could show that cytotoxicity depends on BG composition, BG concentration, particle size, and surface properties. Together with the known osteogenic properties of BGs, our data suggest that BGs represent promising candidates for the treatment of GCTB patients in order to reduce tumor recurrence with simultaneous enhancement of bone regeneration. Since 45S5-BG is already approved by the food and drug administration (FDA) for clinical applications and has already been implanted in >1.5 million patients for bone and dental repair purposes, a transfer of our approach into the clinic might be conceivable very quickly. Before this step, however, future studies addressing the effects of the BG composition and physical parameters such as fragment size and surface properties on BG-mediated cytotoxicity are needed, which will potentially enable a further increase in the effectiveness of this new therapeutic option for GCTB treatment.

## Figures and Tables

**Table 1:** Chemical composition of BGs used in this study in mol%.

BG Name	SiO_2_	CaO	Na_2_O	P_2_O_5_	K_2_O	ZnO	MgO	B_2_O_3_
45S5	46.14	26.91	24.35	2.60	-	-	-	-
ICIE16	49.46	36.27	6.6	1.07	6.6	-	-	-
3Zn	49.46	33.27	6.6	1.07	6.6	3.0	-	-

## Data Availability

All data generated during this study are included in this article.
